# Blood Plasma Metabolites in Diabetes-Associated Chronic Kidney Disease: A Focus on Lipid Profiles and Cardiovascular Risk

**DOI:** 10.3389/fnut.2022.821209

**Published:** 2022-02-28

**Authors:** Ashani Lecamwasam, Toby Mansell, Elif I. Ekinci, Richard Saffery, Karen M. Dwyer

**Affiliations:** ^1^Epigenetics Research, Murdoch Children's Research Institute, Melbourne, VIC, Australia; ^2^Department of Endocrinology, Austin Health, Melbourne, VIC, Australia; ^3^Faculty of Health, School of Medicine, Deakin University, Geelong, VIC, Australia; ^4^Department of Paediatrics, University of Melbourne, Melbourne, VIC, Australia; ^5^Department of Medicine, University of Melbourne, Melbourne, VIC, Australia

**Keywords:** chronic kidney disease, diabetes mellitus, metabolomics, cardiovascular disease (CVD), lipid metabolites

## Abstract

**Background:**

We investigated a cross-sectional metabolomic analysis of plasma and urine of patients with early and late stage diabetes associated chronic kidney disease (CKD), inclusive of stages 1–5 CKD, to identify potential metabolomic profiles between the two groups.

**Methods:**

This cross-sectional study recruited 119 adults. Metabolomic biomarkers were quantified in 119 non-fasted plasma and 57 urine samples using a high-throughput proton Nuclear Magnetic Resonance platform. Analyses were conducted using R with the ggforestplot package. Linear regression models were minimally adjusted for age, sex, and body mass index and *p*-values were adjusted for multiple comparisons using the Benjamini-Hockberg method with a false discovery rate of 0.05.

**Results:**

Apolipoprotein A1 concentration (ApoA1) was reduced (adj. *p* = 0.04) and apolipoprotein B/apolipoprotein A1 ratio (ApoB/ApoA1) was increased (adj. *p* = 0.04) in late CKD compared with early CKD. Low-density lipoprotein triglyceride (LDL-TG) had an increased concentration (adj. *p* = 0.01), while concentrations of high-density lipoprotein cholesterol (HDL-C) were reduced (adj. *p* = 0.04) in late CKD compared to early stages of disease.

**Conclusion:**

Our results highlight the presence of abnormal lipid metabolism namely significant reduction in the protective ApoA1 and significant increase in atherogenic ApoB/ApoA1 ratio. The study also demonstrates significantly elevated levels of triglyceride-rich lipoproteins such as LDL-TG. We illustrate the significant reduction in protective HDL-C in individuals with diabetic CKD. It explores a detailed plasma lipid profile that significantly differentiates between the late and early CKD groups as well as each CKD stage. The study of complex metabolite profiles may provide additional data required to enable more specific cardiovascular risk stratification.

## Introduction

Diabetes and its associated complications have reached pandemic proportions, with a global prevalence of 537 million adults with diabetes (1). Unfortunately, numbers are predicted to rise to 783 million adults by 2045 (1). More than 40% of people with type 2 diabetes will ultimately develop chronic kidney disease (CKD), the leading cause of end stage kidney disease (ESKD) requiring renal replacement therapy (2). Diabetes-associated CKD is associated with an excess all-cause and cardiovascular mortality and is one of the most important causes of health care expenditure, disability, economic loss and mortality (3). Cardiovascular disease (CVD) is the leading cause of CKD-associated death, in part due to persistent low-grade inflammation and subsequent atherosclerosis, a result of atherogenic factors that constitute the metabolic syndrome (4, 5) and factors relating to the metabolic dysregulation of CKD. Diabetes-associated CKD is therefore considered a major global health issue (6).

Metabolomics is the large scale study of small molecules in a sample of biological fluid, such as blood, urine or saliva (7). Metabolites act as functional readouts of underlying physiological processes (8) and are increasingly being used to study kidney function and disease (9, 10).

The global scale and burden of diabetic-associated CKD, particularly its contribution to CVD, prompted us to explore the metabolomic profile in individuals with late CKD stage (Stage 3b−5) and early stages of the disease (Stage 1–3a). We also aimed to identify any potential metabolomic signatures that may have future utility for the early identification of CKD individuals at increased CVD risk.

## Materials and Methods

### Participants

One hundred and twenty one adults with diabetes and CKD stages 1–5 at the time of their outpatient endocrinology clinic visit were recruited based on serum estimated glomerular filtration rate (eGFR as determined by Chronic Kidney Disease Epidemiology Collaboration (CKD-EPI) (11) with or without albuminuria. Where available, albuminuria was evaluated by measuring urinary albumin-to-creatinine ratio (ACR) in a spot urine sample. Microalbuminuria was defined as an ACR of 30–300 mg/g and macroalbuminuria defined as an ACR >300 mg/g (12). Participants qualified for inclusion if they were aged >18 years and were individuals with diabetes who had CKD for at least 3 months. Participants were divided into 2 distinct groups: “early CKD” and “late CKD”. The early diabetic CKD group was defined as participants with diabetes who had stage 1, 2 or 3a CKD (*n* = 83), while the late diabetic CKD group was defined as participants with diabetes who had stage 3b, 4 or 5 CKD (*n* = 38). Participants were excluded if they were <18 years, had acute kidney injury, history of renal transplant, a single kidney, diabetes secondary to pancreatic pathology, steroid medication-induced diabetes, presence of non-diabetic kidney disease, active drug or heavy alcohol use, an active malignancy within the past 5 years, inflammatory bowel disease, were pregnant or breastfeeding or who had a Body Mass Index (BMI) < 20 or > 40 (13).

Data collection occurred only at one time point and included blood pressure, medical comorbidities, duration of diabetes, stage of CKD and associated complications, medications and pathology results. Anthropometric data was collected on the day of the clinic visit while the remainder of the patient's information was gathered from Austin Health's electronic medical record.

### Blood Collection

5 mL of peripheral blood was collected in an Ethylenediaminetetraacetic acid (EDTA) anticoagulant tube and centrifuged at 3500 rcf at 4°C. The resultant plasma was separated into 0.5 ml aliquots. Samples were processed within 2 h of collection and aliquots stored at−80°C until thawed for metabolomic profiling.

### Urine Collection

A spot urine collection was transported to the laboratory within 24–48 h of collection, centrifuged at 3500 rcf at 4°C, aliquoted into 5 ml tubes and stored at −80°C within 30 min of processing.

The research protocols that the authors developed for blood and urine collection have already been published (13). The methods outlined in the published protocol allow for replication studies.

### Metabolomics Data and Statistical Analysis

#### Metabolomics Data

After quality control, metabolomic measures were quantified in 119 non-fasted plasma and 57 urine samples using Nuclear Magnetic Resonance (NMR) metabolomics (14, 15). A total of 225 metabolic measures within 14 subclasses were measured in plasma, while the urine NMR panel quantified 54 metabolites.

#### Statistical Analysis

Analyses were conducted using R (version 3.5.3) with the ggforestplot (v0.0.2) and WGCNA (v1.69) packages. The concentrations of all metabolomic measures were log-transformed and scaled to a standard distribution (SD units) to allow for comparison between metabolites across a range of concentrations. Principal-component analysis was performed, with pairwise Pearson's correlations calculated for each of the clinical and lifestyle variables with the initial five principal components. Scatterplots of the first and second principal components were used to visualize differences in the variance of samples on the basis of sex, CKD group (early/late), or CKD stage 1–5 (on a linear scale).

Linear regression models were used to estimate the association between CKD group or CKD stage as the exposure and each metabolomic measure as the outcome. Linear regression models were minimally adjusted for age, sex, and BMI. Potential confounders were iteratively added to models and retained in the final model if they altered any estimated coefficients by more than 10% (16). Linear regression model *p*-values were adjusted for multiple comparisons using the Benjamini-Hochberg method (17) with a false discovery rate (FDR) of 5%.

## Results

### Clinical and Biochemical Characteristics

**Figure 1:**
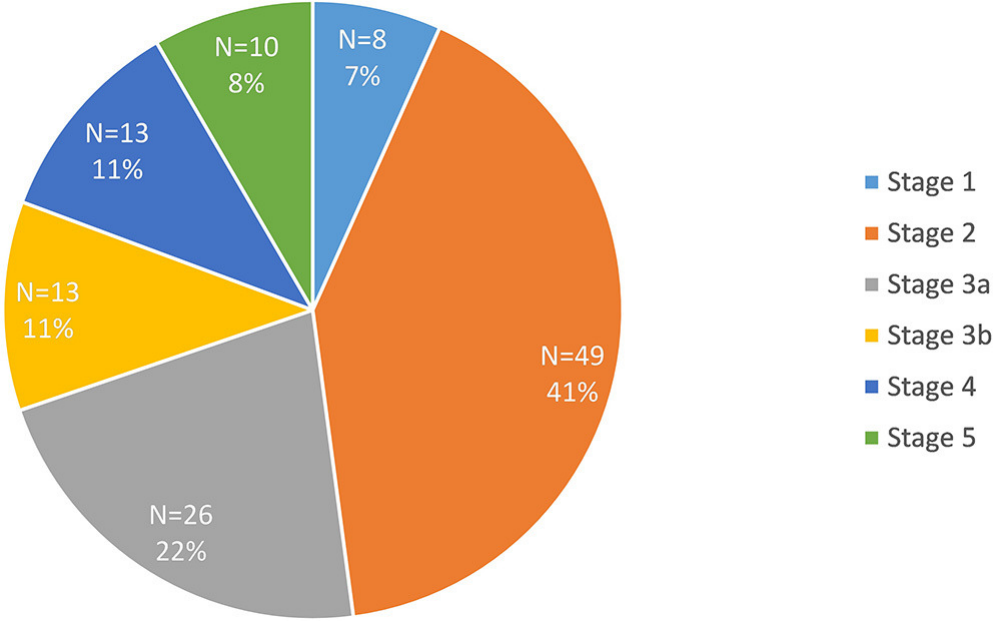
Proportion of participants in each stage of chronic kidney disease (CKD).

Data were available for 119 participants after quality control. The clinical and biochemical characteristics of this sample are shown in Table 1. Type 2 diabetes was present in the majority of participants (*n* = 99). There were 83 participants in the early diabetes-associated CKD group (stages 1, 2 and 3a; mean eGFR 61 ml/min/1.73 m^2^, range 45 to 91 ml/min/1.73 m^2^), and 36 participants in the late diabetic CKD group (stages 3b, 4 and 5; mean eGFR of 24 ml/min/1.73 m^2^, range 4 to 43 ml/min/1.73 m^2^). This distinction between early and late groups was made in light of the marked increase in death, cardiovascular events and hospitalizations observed as eGFR falls below 45 ml/min/1.73 m^2^ (18). The mean age of 72 years in the late CKD group was significantly older when compared with the younger mean age in the early CKD group of 66 years (*p* < 0.05). There was a lower proportion of males (44%) in the late group compared with 60% males in the early group. The proportion of the 119 recruited participants in each stage of CKD is illustrated in Figure 1. Similar participant results are observed in a separate study (19) by the authors as the same patient group was used for both studies.

**Table 1:** Clinical and biochemical characteristics of participants.

**Patient characteristics**	**Mean early CKD**	**SD or %**	**Mean late CKD**	**SD or %**	***P*-value**
	**(Group 1)**		**(Group 2)**		
Age (yrs)	66.14	11.5	72.00	11.5	0.01
Male	50	60%	16.00	44%	0.16
Type of diabetes					0.24
Type 1	15	18%	3	8%	
Type 2	66	80%	33	92%	
LADA	2	2%	0	0%	
Duration of diabetes (yrs)	18.71	11.0	33.00	11.2	0.11
Hypertension	65	78%	34.00	94%	0.06
Diabetic retinopathy	32	39%	15.00	42%	0.91
Cardiovascular disease	30	36%	15.00	42%	0.72
Stroke/Transient Ischaemic Attack (TIA)	10	12%	4.00	11%	1.00
Peripheral vascular disease	12	14%	10.00	28%	0.14
Dyslipidemia	66	80%	31.00	86%	0.55
Depression	16	19%	4.00	11%	0.41
Smoking status					0.51
Non-smoker	46	55%	24	67%	
Ex-smoker	30	36%	10	28%	
Current-smoker	7	8%	2	6%	
BMI (kg/m^2^)	29.44	7.9	28.53	7.9	0.58
SBP (mmHg)	109.58	49.7	121.09	50.0	0.26
DBP (mmHg)	63.38	27.2	66.20	20.1	0.58
Hb (g/L)	108.60	52.3	88.75	53.7	0.06
eGFR (ml/min/1.73 m^2^)	61.17	22.8	23.89	12.0	<0.001
HbA1c (%)	7.51	1.8	7.66	1.7	0.69
TC (mmol/L)	4.00	1.1	3.74	1.0	0.25
LDL (mmol/L)	1.89	0.9	1.78	0.9	0.54

We used the Principal Component Analysis (PCA) method to transform the large set of variables in the metabolomic data set into a smaller set still containing all the relevant information. This analysis did not discriminate between early and late CKD groups (Supplementary Figure 1). There was also no discrimination between genders (Supplementary Figure 2).

**Figure 2:**
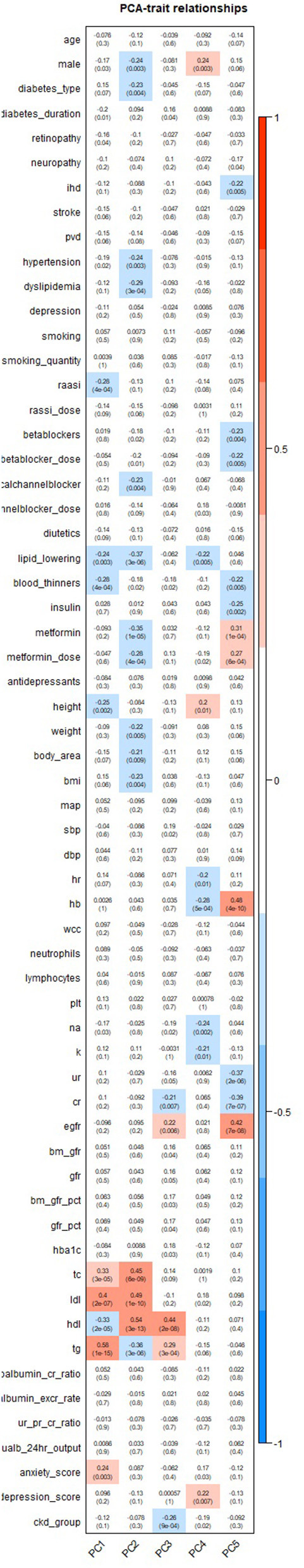
Heatmap illustrating the correlation of principal components with clinical and biochemical characteristics.

The heatmap (Figure 2) demonstrated the correlation of the principal components to the clinical and biochemical variables in our participants. Principal component 1 (PC1) was most strongly correlated with lipid measures of total cholesterol (TC), low-density lipoproteins (LDL), high-density lipoproteins (HDL) and triglycerides (TG). Principal component 2 (PC2) was also most strongly correlated with the same lipid measures.

**Figure 3:**
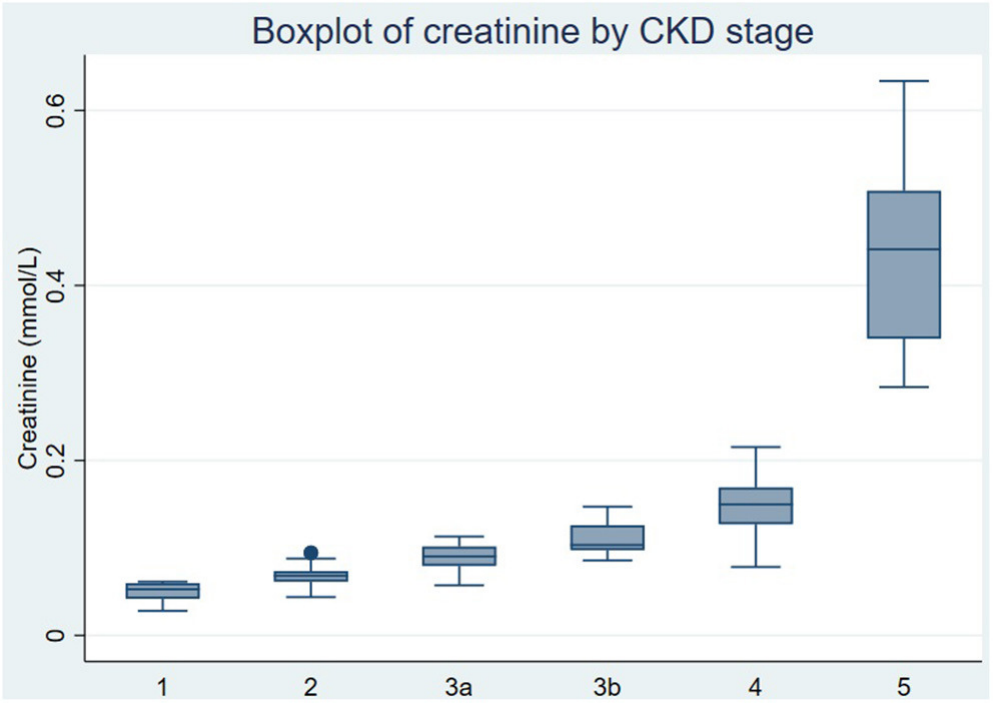
Boxplot of creatinine by chronic kidney disease (CKD) stage.

**Figure 4:**
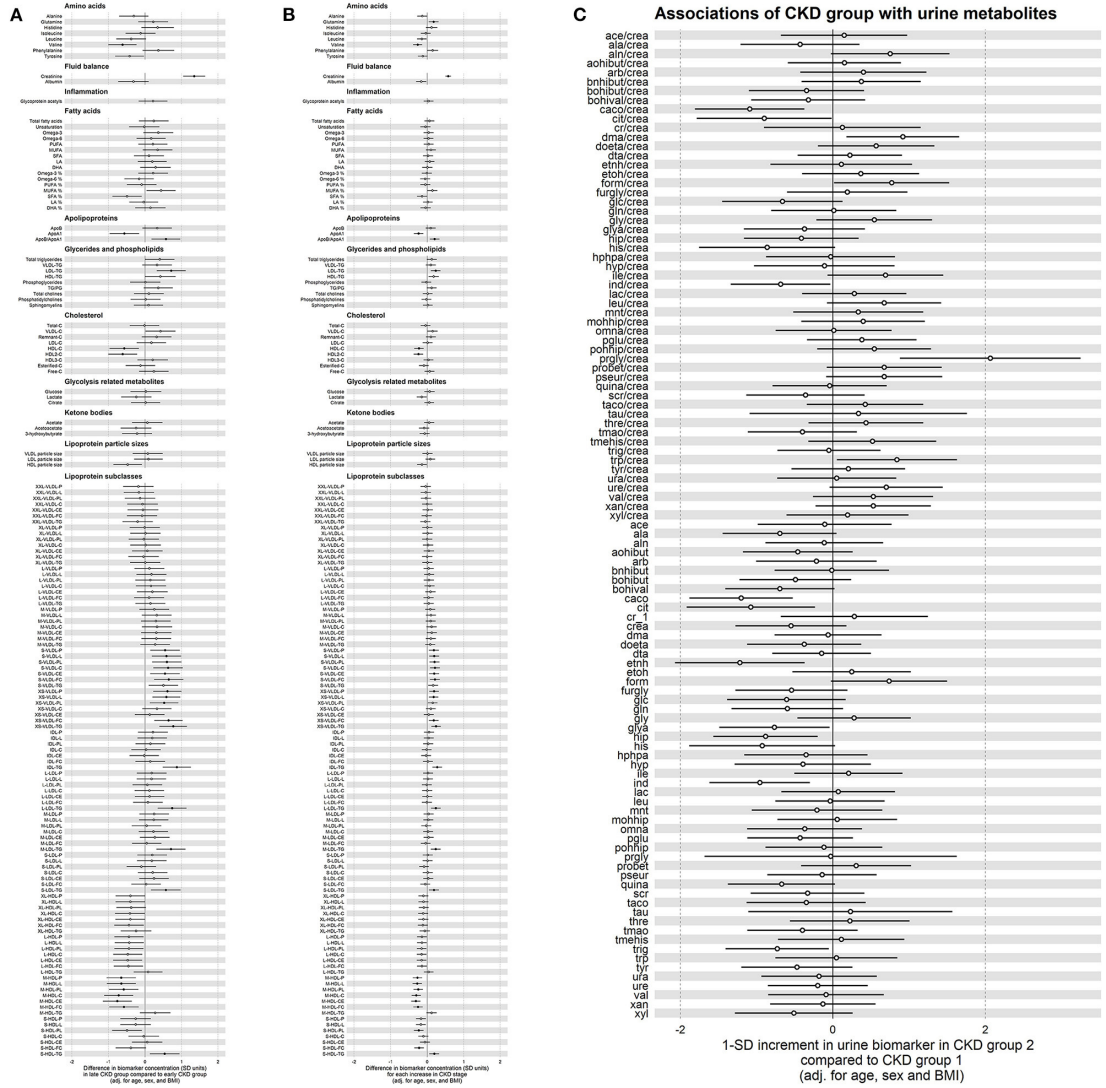
**(A)** Forest plot comparing plasma metabolite concentrations between late and early chronic kidney disease (CKD) group. Black colored in circle signifies statistical significance. Values to the right of the midline are present in higher concentrations in late compared to early CKD group. Values to the left of the midline are present in lower concentrations in late compared to early CKD group. Model has been adjusted for age, sex and body mass index (BMI). **(B)** Forest plot comparing plasma metabolite concentrations at each stage of chronic kidney disease (CKD) when compared with the previous stage of CKD. Black colored in circle signifies statistical significance. Values to the right of the midline are present in higher concentrations at each stage of CKD when compared to previous stage. Values to the left of the midline are present in lower concentrations at each stage of CKD when compared to previous CKD stage. Model has been adjusted for age, sex and body mass index (BMI). **(C)** Forest plot comparing urine metabolite concentrations between late and early chronic kidney disease (CKD) group. White circle signifies no statistical significance. Values to the right of the midline are present in higher concentrations in late compared to early CKD group. Values to the left of the midline are present in lower concentrations in late compared to early CKD group. Model has been adjusted for age, sex and body mass index (BMI).

A significant and expected rise in creatinine was observed in the late CKD group compared to the early CKD group and across each of the stages of CKD (Figures 4A,B). Mean creatinine levels increased from 59.9 umol/L in stage 1 CKD to 194 umol/L in stage 4 CKD in our patient study (Figure 3) and to 534.6 umol/L in stage 5 CKD. The rise in creatinine in ESKD was depicted by a higher median creatinine with a larger variation within the 25^th^-75^th^ quartile as seen in Figure 3.

### Metabolomic Variation Associated With Transition From Late to Early Stage CKD

After adjustment for age, gender and Body Mass Index (BMI), at least 7 metabolites within 6 classes were consistently and significantly associated with varying kidney function. Comparing late to early CKD groups (Figure 4A) there were lower concentrations of the metabolite valine in the amino acid class (Supplementary Table 1A, adj. *p* < 0.05) and as expected, significantly higher creatinine concentrations in late compared to early CKD (Supplementary Table 1A, adj. *p* < 0.001). The apolipoprotein class revealed significantly lower concentrations of protective apolipoprotein A1 (ApoA1, Supplementary Table 1A, adj. *p* < 0.05) and higher concentrations of atherogenic apolipoprotein B/apolipoprotein A1 ratio (ApoB/ApoA1, Supplementary Table 1A, adj. *p* < 0.05) in late compared with early CKD. The glycerides and phospholipids class demonstrated significantly higher concentrations of pathogenic low-density lipoprotein triglyceride (LDL-TG, Supplementary Table 1A, adj. *p* < 0.05) in late compared to early CKD. Cardioprotective high-density lipoprotein cholesterol (HDL-C) and HDL2-C concentrations were significantly lower in late compared to early CKD (Supplementary Table 1A, adj. *p* < 0.05). The lipoprotein subclasses showed statistically significant higher concentrations in a variety of pathogenic small very-low-density lipoproteins (S-VLDL) in late compared to early CKD including small very-low-density lipoprotein cholesterol (S-VLDL-C, Supplementary Table 1A, adj. *p* < 0.05) and very small very-low-density lipoprotein triglyceride (XS-VLDL-TG, Supplementary Table 1A, adj. *p* < 0.05). Significantly higher concentrations were also observed in intermediate-density lipoprotein triglyceride (IDL-TG, Supplementary Table 1A, adj. *p* < 0.005) and lower concentrations of a variety of medium high-density lipoprotein (M-HDL), including total cholesterol in medium high-density lipoprotein (M-HDL-C, Supplementary Table 1A, adj. *p* < 0.05) in late compared to early CKD. These differential metabolite profiles between late and early CKD are illustrated in Figure 4A and Table 2.

**Table 2:** Summary table of differential plasma metabolites and their concentrations.

**Class**	**Trait/metabolite**	**Trait/metabolite**
	**Low concentration**	**High concentration**
Amino acid	Valine	
Fluid balance		Creatinine
Apolipoprotein	ApoA1	ApoB/ApoA1
Glycerides and phospholipids		LDL-TG
Cholesterol	HDL-C, HDL2-C	
Lipoprotein subclass	M-HDL-C	S-VLDL-C
		XS-VLDL-TG
		IDL-TG

Evaluating progressive diabetes-associated CKD by stages (1–5), (Figure 4B, Table 2) we observed similar significant patterns of metabolite concentrations. Additionally, there was modest evidence for the amino acid glutamine having higher concentrations with progressive CKD stages, though this was not significant after correction for multiple testing (adj. *p* = 0.06) (Figure 4B, Supplementary Table 1B).

The metabolite Glycoprotein acetyls (GlycA), is a biomarker of chronic inflammation. Surprisingly, there was no evidence of significantly higher concentrations of GlycA, in late CKD compared with early CKD or with progressive CKD stage (Figures 4A,B, Supplementary Tables 1A,B).

### Urine Metabolites in Early and Late Diabetes-Associated CKD Groups

In contrast to the significant metabolite changes observed in the plasma between late and early CKD, the urine metabolome did not demonstrate significant differences between late and early stages of CKD. Specifically, there were lower ratios of concentration of cis-aconitate to creatinine (caco/crea), citrate to creatinine (cit/crea) and indoxyl sulfate to creatinine (ind/crea) as well as lower concentrations of individual metabolites such as cis-aconitate, citrate, ethanol, glycolic acid, hippurate, indoxyl sulfate and trigoneline in late CKD compared with early CKD, but these differences were all non-significant after adjustment for multiple testing (Figure 4C, Supplementary Table 1C). Similarly, there were non-significant differences of higher ratios of concentration of dimethylamine to creatinine (dma/crea), formate to creatinine (form/crea), propylene glycol to creatinine (prgly/crea) and tryptophan to creatinine (trp/crea) were present in the urine of individuals with late CKD compared with early CKD (Figure 4C, Supplementary Table 1C).

## Discussion

Along the continuum of CKD, from microalbuminuria to end stage kidney disease, the risk of CVD increases exponentially (20). This study is the first to apply NMR-based metabolomics to both plasma and urine from a group of individuals with predominantly type 2 diabetes-associated chronic kidney disease, comparing late CKD with early CKD across all chronic kidney disease stages. Specifically, this study examined the lipid profile inclusive of apolipoproteins and lipoprotein subclasses and highlights a potential role of dyslipidaemia in relation to cardiovascular risk (21).

### Plasma Metabolome

Validation of this NMR platform was confirmed by the step-wise increment in creatinine across stages 1–4 of diabetes-associated CKD. There was a marked increase in creatinine concentrations from CKD stage 4 to 5 (Figure 3) consistent with the exponential relationship between serum creatinine and eGFR and CKD stage.

#### Glycerides, Apolipoproteins and Lipoprotein Subclasses

Individuals with CKD have a high prevalence of hypertriglyceridemia (22), a consequence of increased production and reduced clearance of triglyceride-rich lipoproteins (TGRL) (23). Atherogenic triglyceride-rich lipoproteins comprise very-low-density lipoproteins (VLDL), chylomicrons and their remnants, intermediate-density lipoprotein (IDL), low-density lipoprotein (LDL) and lipoprotein(a) (24). ApoB is a large protein that is a component of all the atherogenic lipoproteins and provides structural integrity (24), whereas apolipoprotein A (ApoA1) is a component of high-density lipoproteins (HDL) which is anti-atherogenic (25). ApoB and ApoA1 can be directly measured in the blood and have been internationally standardized according to the World Health Organization and the International Federation of Clinical Chemistry (WHO-IFCC) (26).

In this study, we observed significantly higher concentrations of intermediate-density lipoprotein triglyceride (IDL-TG), atherogenic small very-low-density lipoprotein cholesterol (S-VLDL-C) and very small very-low-density lipoprotein triglyceride (XS-VLDL-TG) in late CKD compared to early CKD and across all stages of CKD (Figures 4A,B). Other atherogenic TGRLs included significantly higher concentrations of low-density lipoprotein triglyceride (LDL-TG) in late compared to early CKD as well as in every increment of CKD stage. These atherogenic TGRLs contribute to the progression of atherosclerosis and cardiovascular disease via intimal cholesterol deposition as well as being involved in enhancing proinflammatory, proapoptotic and procoagulant pathways (27). Evidence suggests that the sum of the total cholesterol carried by these atherogenic lipoproteins provides a better indication of cardiovascular risk than LDL-C, particularly in CKD patients with hypertriglyceridemia (27).

The ApoB/A1 ratio in our study was significantly higher in late CKD compared with the early CKD group and across progressive CKD stages (Figures 4A,B). These data are consistent with the known pathogenic processes that underpin progressive glomerular and interstitial lesions and kidney dysfunction (28). The ratio of ApoB/A1 is a well-studied risk factor for cardiovascular disease (29). The large Swedish Apolipoprotein-related MOrtality RISk (AMORIS) study measured ApoB and ApoA1 in more than 175,000 individuals prospectively followed for up to 25 years (30). The AMORIS study found a strong direct relationship between ApoB and risk of myocardial infarction (MI) and an indirect inverse relationship between ApoA1 and risk of MI. Interestingly, ApoB was a stronger risk factor than LDL-C, especially at low values of LDL-C (30). INTERHEART, was another example of a large case-control study that compared participants with a first MI to controls from 52 countries matched for age, gender and ethnicity (31). The results of this study showed that the ApoB/ApoA1 ratio in both sexes, was the strongest and most prevalent risk factor for MI when compared to other lipid measures and traditional risk factors (29, 31). The higher ApoB/ApoA1 ratio seen in our study of patients in late CKD compared to early CKD, is relevant given the magnified cardiovascular risk associated with progressive diabetes-associated CKD (18). The ATTICA study, investigated the risk stratification of apolipoprotein B, apolipoprotein A1 and the ApoB/ApoA1 ratio, in a random sample of adults, with an absence of cardiovascular disease, and showed that, using the area under the Receiver Operation Characteristic (ROC) curve, ApoB/ApoA1 was the best diagnostic marker of metabolic syndrome (32). Given the significant role metabolic syndrome plays in cardiovascular risk and most participants in our study demonstrate the metabolic syndrome, the use of ApoB/ApoA1 is most germane and adds more evidence for its use in the clinical setting.

In clinical practice, the more common measure of cardiovascular risk in the general population are LDL cholesterol concentrations, which are paradoxically not raised in individuals with CKD (22). This is in contrast to the significantly higher concentrations of TGRLs that exists with progressive kidney dysfunction, which is demonstrated in our study results. Our results also demonstrate that LDL cholesterol did not differentiate between the late CKD group compared with the early CKD group (Figure 4A) or across progressive CKD stages (Figure 4B). Another common clinical measure is the serum cholesterol assay, however, in individuals with CKD, it is generally within the normal range (33) and did not increase in this study (Figures 4A,B). Unfortunately, there is a paradoxical association between these low-normal serum cholesterol levels and high mortality in individuals with CKD. This anomaly is due to cardiovascular complications that arise due to increased systemic inflammation and oxidative stress which has the potential to increase oxidized LDL cholesterol levels without an increase in LDL cholesterol in CKD patients (34). It is difficult to shift the well-documented LDL-paradigm and convince committees and clinicians to accept that Apo ratios play a significant role as cardiovascular risk predictors and that these parameters need to be measured and taken into the clinical decision-making process in this group of susceptible individuals. This is reflected in the absence of an acknowledgment of apolipoprotein measurements in the most recent Kidney Disease: Improving Global Outcomes (KDIGO) lipid management guidelines (35).

The clinical implications of lipid profiles in individuals with diabetes and CKD have potential ramifications on patient management. There have been reports of novel TGRL lowering specific therapies, such as evinacumab, an angiopoietin-like protein 3 antibody that can reduce triglyceride levels by up to 70% (36). There may be a potential benefit in using these novel drug therapies beyond the standard of treatment with statins and/or cholesterol absorption inhibitors (ezetimibe) as per the current guidelines set out by KDIGO on lipid management in chronic kidney disease (37).

High-density cholesterol (HDL) is known to show anti-oxidant and anti-inflammatory characteristics as well as reduce the monocyte infiltration in artery intimal walls and thereby hinder the atherosclerotic process (34). Kidney impairment causes monocytosis and monocyte activation, which is an additional risk factor for the accelerated atherosclerosis in CKD (38). Furthermore, in individuals with CKD, there is HDL cholesterol deficiency and impaired HDL metabolism (39), which results in impaired anti-oxidant effects leading to increased oxidized LDL cholesterol formation, atherosclerotic risk and mortality (40). The results from our study underscore impaired HDL metabolism. Comparing late CKD with early CKD, as well as for every increment to CKD stage there is a significantly lower concentration of HDL-C as well as HDL-2 cholesterol (Figures 4A,B). This metabolomic pattern illustrates the dyslipidaemia and cardiovascular risk in this group of highly susceptible patients.

#### Amino Acids

The branched chain amino acids (BCAA) valine, isoleucine and leucine play a pivotal role in metabolism as precursors for the synthesis of proteins, fatty acids, regulators of protein turnover and insulin release (41). We found a significantly lower plasma concentration of valine in the late CKD group compared with the early CKD group (Figure 4A) and across stages of CKD (Figure 4B) which was in keeping with results of other studies (42). Interestingly in a study of dialysis patients, plasma isoleucine and leucine were normal except in the malnourished patients, whereas valine was reduced more than would be expected from malnutrition alone (42). Ketoanalogues of amino acids (KA) are nitrogen-free analog supplements of essential amino acids (EAA) such as valine, leucine, isoleucine and phenylalanine (43). In individuals with CKD with impaired nutrition, as reflected by this imbalance of EAA, current evidence suggests that a low protein diet with KA supplementation should be included as part of the clinical recommendations for both the nutritional prevention and metabolic management of CKD (44).

### Urine Metabolome

Urine is often considered a favorable biofluid for analysis as it is easily accessible, collected rapidly, non-invasively and cheaply. It is also chemically complex and this complexity has made it a difficult substrate to fully understand (45). Despite observing a lower concentration of metabolites such as cis-Aconitate, citrate, ethanol, glycolic acid, hippurate, indoxyl sulfate and trigoneline in late CKD compared to early CKD, these metabolites did not reach statistical significance after multiple testing (**Figure 4C**). Similarly, there were higher concentrations of particular metabolite ratios in late CKD compared to early CKD (**Figure 4C**), but these too were non-significant. This may be due to urine being highly reflective of the exposome and in contrast to plasma having greater fluctuations of metabolite levels making it harder to quantify molar concentrations of metabolites in such an unstable biological medium.

## Strengths and Limitations

One of the strengths of this study is its investigation of both plasma and urine samples, using the NMR platform, across the spectrum of diabetes-associated CKD. To the best of our knowledge, it is the only study to evaluate the metabolomic profiles between late and early diabetic CKD as well as at every increment of CKD stage. It further explores a detailed plasma lipid profile that significantly differentiates between the late and early CKD groups as well as each CKD stage. One of the limitations however, is its small sample size, especially the presence of only 36 participants with the late stages (3b−5) diabetic CKD resulting in a potential loss of generalizability and lack of power to detect associations. Notwithstanding it is beneficial to have a larger number in the early group to identify possible metabolomic patterns at an earlier stage. Furthermore, the cross-sectional nature of this study meant patient samples were collected at only one time point and not studied longitudinally. Despite the limitations however, this study has shown some evidence of metabolomic variation in blood in association with diabetic CKD and hence provides the foundation for future directions for testing with a larger and longitudinal patient population.

## Conclusion

This metabolomic analysis, inclusive of plasma and urine, provides novel insight into the metabolome of individuals with diabetes and varying degrees of kidney impairment. Our results highlight the presence of abnormal lipid metabolism namely significantly lower concentrations of the protective ApoA1, HDL-C, HDL-2 cholesterol and significantly higher concentrations of the atherogenic IDL-TG, S-VLDL-C, XS-VLDL-TG, LDL-TG and ApoB/ApoA1 ratio in late compared to early stages of CKD as well as in every increment of CKD stage compared with the previous stage of disease. Significantly, the finding of higher ApoB/ApoA1 ratio in late compared to early CKD reinforces the recommendation to use apolipoproteins, particularly ApoB/ApoA1 as novel tools for predicting lipid-related cardiovascular risk. Perhaps a new approach using the ApoB/ApoA1 paradigm is needed for future cardiovascular risk assessment in this high risk group of individuals, rather than current day clinical “standards” of serum LDL, HDL and cholesterol measures. Further research is needed to validate these findings as well as its potential role as biomarkers in diabetic CKD and CVD risk in longitudinal, large cohort studies.

## Data Availability Statement

All data generated or analyzed during this study are included in this published article and its Supplementary Materials.

## Ethics Statement

The studies involving human participants were reviewed and approved by the Human Research Ethics Committee of Austin Health, Victoria, Australia (HREC/17/Austin/166) and the Human Research Ethics Committee of Deakin University, Australia. The procedures followed were in accordance with the Helsinki Declaration of 1975, as revised in 2013. The patients/participants provided their written informed consent to participate in this study.

## Author Contributions

AL, EE, RS, and KD designed the study. AL processed initial samples. TM analyzed the data and made the figures. AL, TM, EE, RS, and KD drafted and revised the paper. All authors approved the final version of the manuscript.

## Funding

AL was supported by a Deakin University School of Medicine Postgraduate Research Scholarship. EE was supported by the Sir Edward Weary Dunlop Medical Research Foundation Principal Research Fellowship at the University of Melbourne and the NHMRC research funding. EE's institution has received research funding from Novo Nordisk, Bayer, Sanofi, and Dimerix.

## Conflict of Interest

The authors declare that the research was conducted in the absence of any commercial or financial relationships that could be construed as a potential conflict of interest.

## Publisher's Note

All claims expressed in this article are solely those of the authors and do not necessarily represent those of their affiliated organizations, or those of the publisher, the editors and the reviewers. Any product that may be evaluated in this article, or claim that may be made by its manufacturer, is not guaranteed or endorsed by the publisher.

## Abbreviations

ApoA1: Apolipoprotein A1
ApoB: Apolipoprotein B
BCAA: Branched-chain amino acids
BMI: Body mass index
CKD: Chronic kidney disease
CVD: Cardiovascular disease
EDTA: Ethylenediaminetetraacetic acid
Egfr: Estimated glomerular filtration rate
ESKD: End-stage kidney disease
FDR: False discovery rate
GC-MS: Gas chromatography with mass spectrometry
GlycA: Glycoprotein acetyls
HDL: High-density lipoprotein
HDL-C: High-density lipoprotein-cholesterol
HREC: Human Research Ethics Committee
IDL: Intermediate-density lipoprotein
IDL-TG: Intermediate-density lipoprotein-triglyceride
LC-MS: Liquid chromatography with mass spectrometry
LDL: Low-density lipoprotein
LDL-C: Low-density lipoprotein- cholesterol
LDL-TG: Low-density lipoprotein-triglyceride
MCRI: Murdoch Children's Research Institute
M-HDL-C: Medium high-density lipoprotein cholesterol
NMR: Nuclear magnetic resonance
PC: Principal component
PCA: Principal component analysis
S-VLDL-C: Small- very low-density lipoprotein-cholesterol
TC: Total cholesterol
TG: Triglyceride
TGRL: Triglyceride-rich lipoprotein
VLDL: Very low-density lipoprotein
WHO: World Health Organization
XS-VLDL-TG: Very small- very low-density lipoprotein-triglyceride.
