# Supplementary figures and images for: Blood Plasma Metabolites in Diabetes-Associated Chronic Kidney Disease: A Focus on Lipid Profiles and Cardiovascular Risk
Source: Front Nutr. 2022 Feb 28;9:821209. doi: 10.3389/fnut.2022.821209 (PMC8918794; doi:10.3389/fnut.2022.821209)

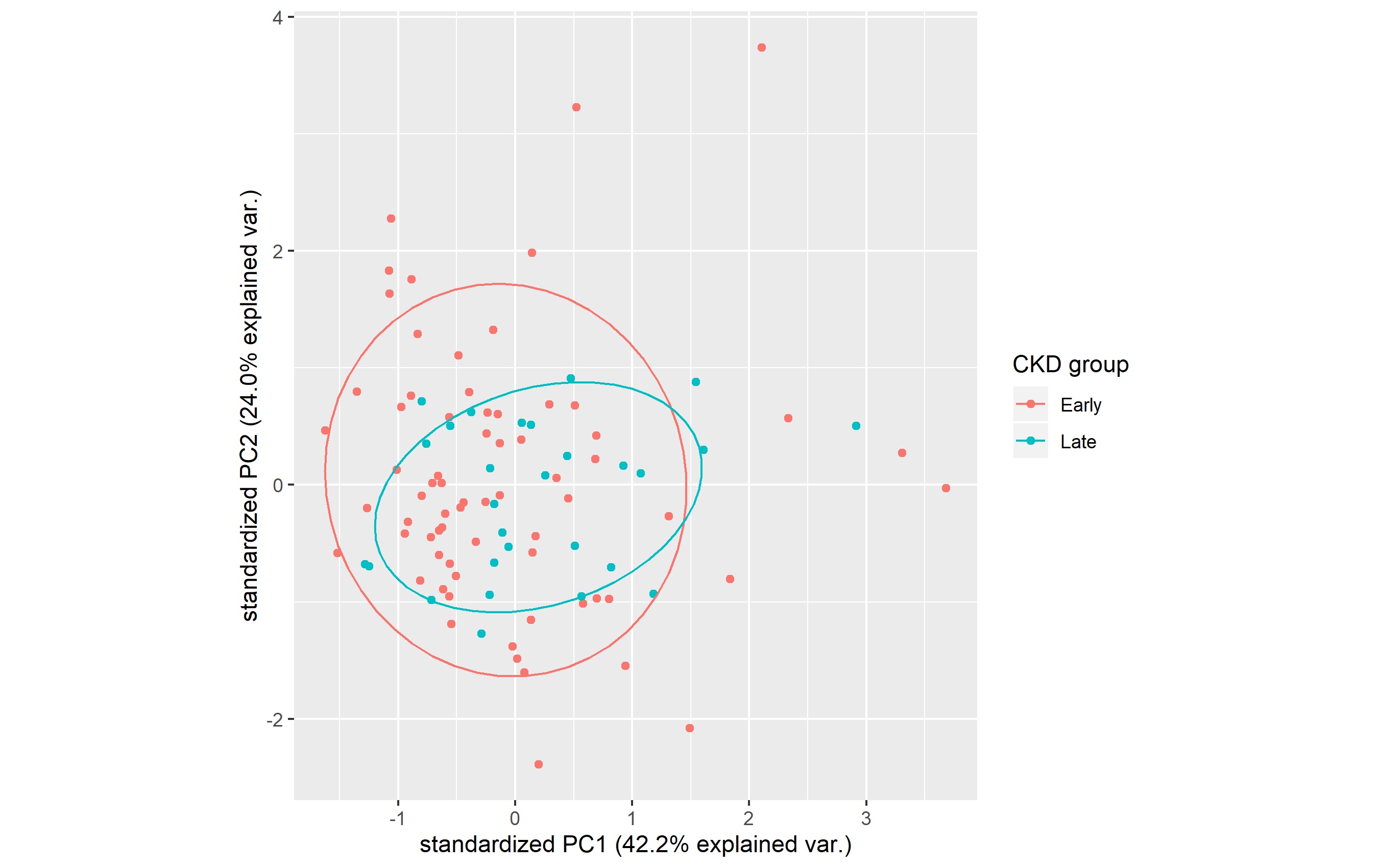

Supplement: Supplementary file 1 [file Image_1.JPEG]

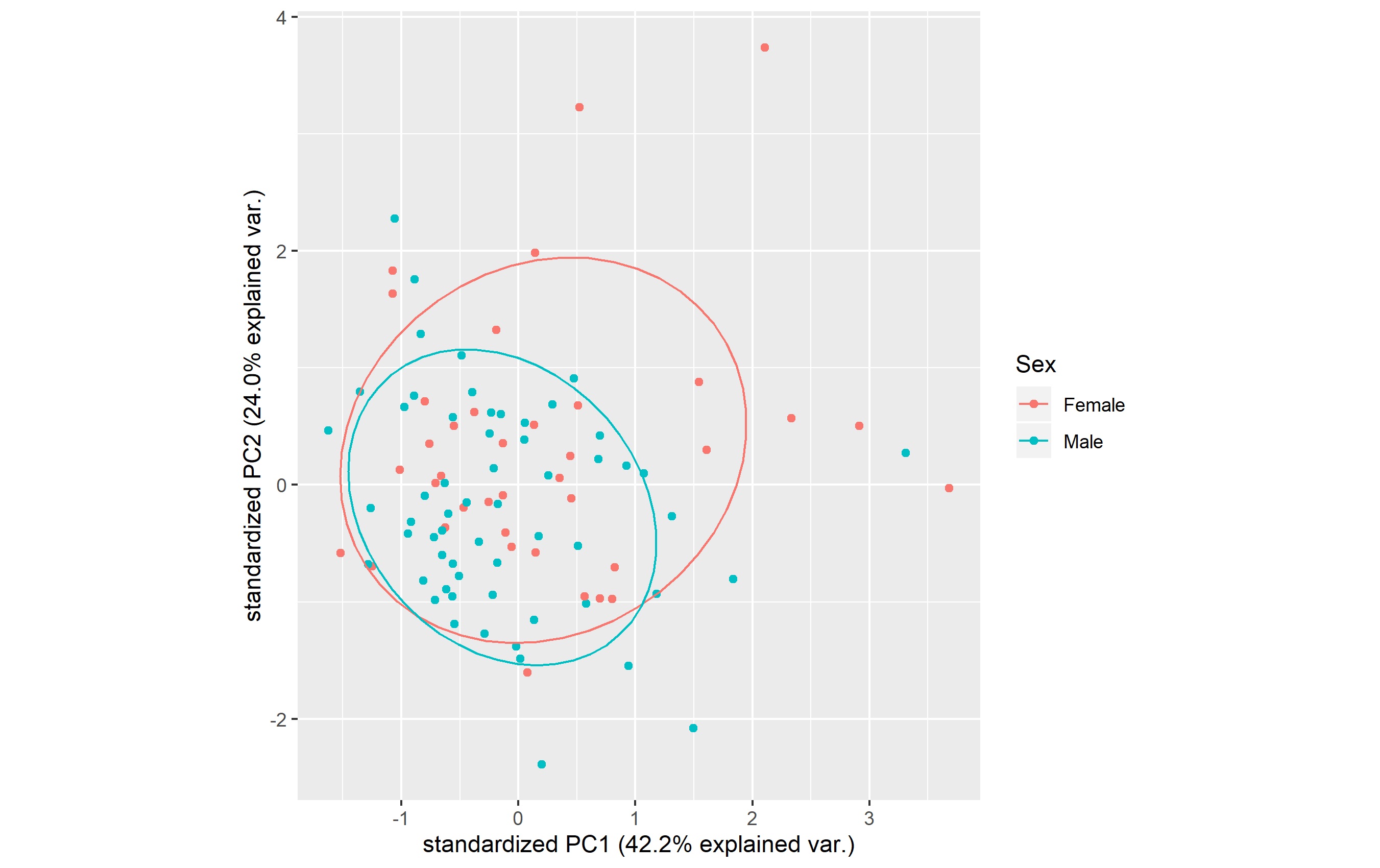

Supplement: Supplementary file 2 [file Image_2.JPEG]

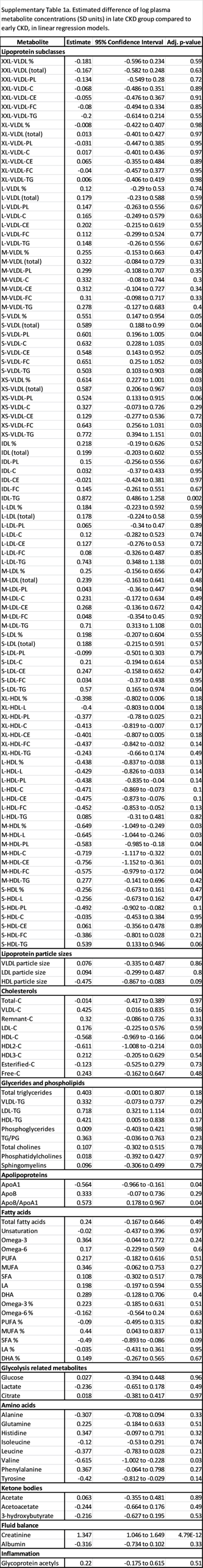

Supplement: Supplementary file 3 [file Image_3.JPEG]

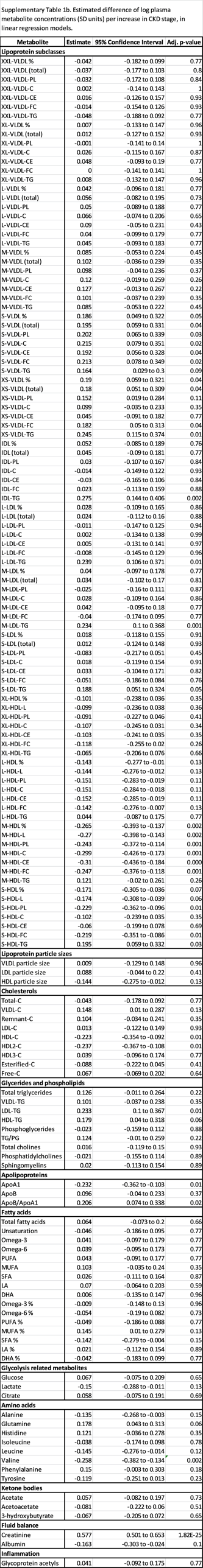

Supplement: Supplementary file 4 [file Image_4.JPEG]

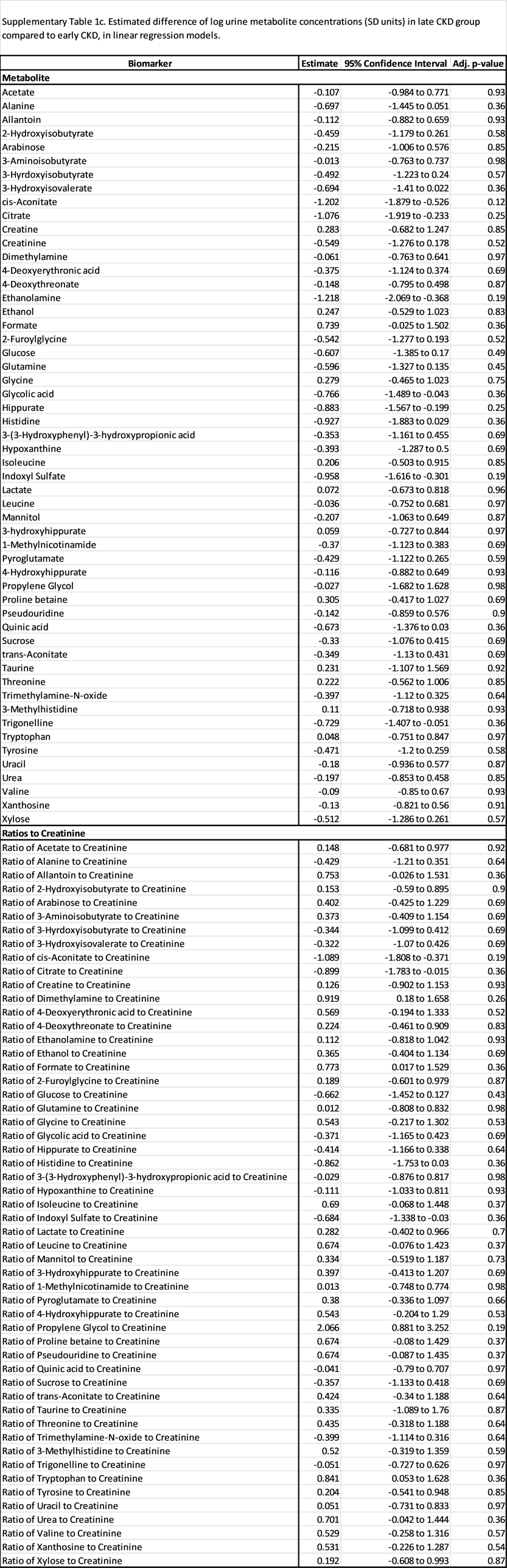

Supplement: Supplementary file 5 [file Image_5.JPEG]
